# Supplementary material for: The arabidopsis WAVE/SCAR protein BRICK1 associates with cell edges and plasmodesmata
Source: PLoS One. 2025 Sep 8;20(9):e0325015. doi: 10.1371/journal.pone.0325015 (PMC12416715; doi:10.1371/journal.pone.0325015)
Supplement: Attachment — Submitted filename: response to reviewers.docx [file pone.0325015.s003.docx]

# Editor’s comments

## Major comments

A. Figure 1. You are claiming that figure 1 establishes that brick1 localizes to the outer cell wall edges. But this figure is not convincing in that regard. In Figure 1B, cell wall staining vanishes before the inner edges are reached. How do you know that the brick1 staining also did not vanish for the same reason (i.e., imaging parameters not absence of protein)? In the other cell types, there is no attempt to show inner vs outer cell edges. The paper would be much strengthened by some z-stacks going from outer periclinal wall to inner periclinal wall (and if possible beyond).

Response: Thank you. We have improved the figure and added a 3D rotation supplemental movie. A close look shows that most walls show little attenuation, or when signal does decrease with depth, the drop is not as sharp as seen with outer edge-localized BRK1-YFP. Similarly, by comparing the relative BRK1-YFP signal along the anticlinal edges, there is relatively little attenuation of signal (see also figure 1h, root epidermal cells). In addition to these internal comparisons, in many of our images, not all cells in the sample are angled perfectly normal to the optical z-axis (such as near the edges of a rounded organ), providing a true tilted view of the cells. In these cells, the periclinal signal at the outer edge still shows strong enrichment. For examples, see the un-tilted z-projections of the curved leaf surface in figure 1A-C, and the un-tilted z-projection of a lobed pavement cell with slanted walls on its right and bottom side. We added lines to indicate the inner cell edges of these walls. We also provide separate channel images now, which show that FM4-64 signal attenuation is minimal.

B. Along these lines, you claim in the discussion that brick1 is localized to cell wall junctions in mesophyll and that this localization is a first non-epidermal localization for this protein. However, Figure 1G, where this localization is shown, is not presented in the results specifically. Moreover, without a z-stack, I am not convinced that the imaged cell junctions are mesophyll. How do you know?

Response: Thank you for pointing this out. These cells are the first layer of spongy mesophyll. We now mention the mesophyll panel in the results, and we marked intercellular spaces clearly with asterisks.

C. In the discussion (Line 149) you write that brick1 is most evident in mature cells. Where is this shown in the results? A developmental time series (or several) showing young, middle aged, and old organs (hypocotyls? cotyledons? leaves?) would make this point and strengthen the paper.

Response: We were referring specifically to the PD localization of BRK1-YFP. Line 149 says “BRK1-YFP accumulation at PD is most evident in mature cells…” We have clarified what we mean by mature cells in the text (see additional comments below).

D. Finally, in figure 1E and F, and also figure 4, brick1 seems to be preferentially localized to transverse walls (compared to longitudinal walls). This looks striking. I am surprised that you don’t comment on this apparent polarity. If it is real and not an accident of the chosen images then that would be worth documenting in a few figures/experiments.

Response: In elongating/elongated cell types of roots and hypocotyls, such as those shown , BRK1-YFP is indeed enriched at transverse walls compared to radial walls. We now describe this in the results. While it is indeed very interesting, we leave out any speculation in the discussion (although personally I think it has to do with exclusion of microtubules from transverse walls in these cell types, as well as local fortification of these vulnerable regions to keep them from tearing apart lengthwise).

## Minor comments (given in order of occurrence).

1. Please spell out plasmodesmata. Scientists love acronyms but readers hate them. And, writers ought to hate acronyms too, because they convert a word to a symbol. Doing so impedes thought. Given that plasmodesmata are at the heart of the paper, the writer should think about the language carefully. Write with words not symbols. I am of course aware that PD is standard but so is bad writing. Popularity should not justify avoiding clarity.

Response: Done.

1a, Along the lines of promoting clarity I want to add a more speculative suggestion: instead of BRK1, write brick1. The latter is easier to read and looks more handsome on the page. After all, we write actin and not ACTIN. Your protein has a nice name so why not use it? The same can be said for scar/wave. Those are good stout Anglo-Saxon words and look great as such (whereas SCAR/WAVE is an ugly blot). According to arabidopsis lore, genes are supposed to be written in all capital letters; besides that convention differing for other organisms, for the most part here you are writing about proteins. Please consider.

Response: After looking at this carefully, seeing things like brk1-YFP, or brk1-yfp, we choose to stick with convention because most readers of this paper are specialists and any deviation from convention is likely to increase, not decrease confusion or readability.

2. Line 22**.** Change "Arabidopsis" to either *Arabidopsis thaliana* (and then *A. thaliana* after first mention) or arabidopsis. The latter is perfectly correct and would have been the default prior to the advent of molecular biology. A capital letter signifies either a proper name (e.g., Queen Anne's lace) or a genus. Common names are widely taken from the genus (iris, rhododendron) and they are not capitalized or italicized. Unfortunately despite this well established and sensible convention, journals will capitalize Arabidopsis when used as the common name for *A. thaliana*. So the only correct option available to a careful writer is *A. thaliana*.

Response: We have changed it to *A. thaliana*.

3. Line 49. Spell out ‘actin-binding proteins’.

Response: Done.

4. Line 60. You used ‘ADF3’ previously. The convention is to define the acronym at first use. Also while the letters in the acronym are capitalized the letters in what they stand for are not. So it should be written ‘actin-depolymerizing factor 3’. Finally, please consider dropping this acronym altogether.

Response: We dropped the acronym and went with the suggested all-lowercase format with no hyphen before the 3. (Searching the literature found a mix of mostly Actin-Depolymerizing Factor 3 and actin-depolymerizing factor 3).

5. Line 67. Here you write “Arp2/3” but other places you write “ARP2/3”. You should be consistent. While brick and scar and wave, being words, lend themselves to being spelled out, arp is less ‘word like’. I have no problem with arp2/3 but others might.

Response: We have now changed it to be consistently ARP2/3. (Interestingly, again the literature is completely mixed with all caps, first-letter cap, and all lowercase!)

6. Line 74 (and elsewhere). The terminology is confusing. What is a periclinal edge? I think the heading means where the edges between periclinal and anticlinal walls meet. But why not simply ‘outer’ edges? And are you really sure of absnce along an edge between two anticlinal walls?

Response: Thank you for the feedback. “Outer edge” is a great idea, so we tinkered with using outer edge vs. anticlinal edge (as well as a few other combinations variations), but we just couldn’t get it to flow naturally or ease clarity, so we have decided to stick our current periclinal/anticlinal edge terminology, which matches our previously established nomenclature for cell edges (Ambrose et al, Nat Comm 2011, doi: 10.1038/ncomms1444). We have added some extra text to hopefully help clarify.

7. Line 76. Spell out microtubule. And consider writing ‘clasp’ (another good word).

Response: We have now changed MT to microtubule, but kept CLASP, as is convention.

8. Line 78. No reason to capitalize ‘Soseki’. It is not a person.

Response: We checked this one and everybody writes it with all capitals, so we changed it to that, again for consistency with convention and with the literature.

9. Lines 93, 94. I cannot see any solid or dotted lines in Figure 1D.

Response: Thanks, we changed the line color to make it stand out better against our new coloring scheme.

10. Line 95. What does “maturing” mean? Better to give age and rough position. Dark or light grown?

Response: We have changed it to “elongating”. We had used maturing to emphasize the distinction with between young, rapidly expanding cells and “mature” cells, where we see pit fields and the BRK1-YFP plasmodesmatal localization, but elongating is probably clearer in this case. All of our plants are light grown, age 3-7 days after germination, using first true leaves at representative ages. We were generally cautious about simply indicating plant age because in the case of leaves for example, there are always new leaves forming. Generally, for our young leaf images like in figure 1A, these would be the first true leaves when they are still very young and straight, having not yet folded backwards or cupped, and the cells are still rapidly dividing.

11. Line 104 (and elsewhere) spell out propidium iodide.

Response: For this one, we ended up deciding to stick with PI, partially because it is so common that everybody even calls it that in conversations, and partially because it took up too much space on the figure panels, and it was used many times.

12. Line 125 and elsewhere. Do not capitalize aniline blue.

Response: Done.

13. Line 132. Change ‘becomes’ to ‘became’ and ‘remains’ to ‘remained’.

Response: Done.

# Reviewer #1:

The manuscript presents a small but solid piece of descriptive, qualitative work that confirms and extends previous studies (most notably Dyachok et al 2008, Ref. 32), providing additional evidence from hitherto uncharacterized cell types, and, more importantly, presents very high quality image documentation surpassing previous reports.

The only substantial weakness I see is lack of clarity with regard to how did the authors pick BRK1 for detailed characterization. They refer to a "screen to identify proteins that accumulate at cell edges" (l. 75) but do not provide any further information how this screen was conducted. Thus, there is a hole in their story which needs to be repaired - either by incuding the description of this screen (which would strenghten the paper substantially!), or by providing a reference to a paper (or preprint) where their screen is reported.

Response: We thank the reviewer for the constructive comments. For the screen for cell edge proteins, this was actually not a large scale “screen” so to speak, but more of a targeted approach where we requested a dozen or so fluorescent-tagged candidate proteins from colleagues and then checked each for cell edge enrichment. BRK1-YFP was the one we found, and we have reworded the text to reflect this more accurately.

Otherwise there are a couple of less substantial issues that ought to be addressed when revising the manuscript:
1) l. 30-40: The introduction should cite some recent review - there are quite a few (e.g. https://doi.org/10.1111/nph.19666, https://doi.org/10.1093/jxb/erae307).

Response: Thank you, we have now cited these reviews.

2) l. 50 - Myosin VIII at plasmodesmata was found much earlier than 2014, see, e.g., https://doi.org/10.1016/s1065-6995(02)00330-x.

Response: We apologize for the oversite, we now include the suggested reference, along with two early studies from 1998 that used antibodies to animal myosins: (Radford, J. E., & White, R. G. (1998). The Plant Journal, 14(6), 743–750.) and (Blackman, L. M., & Overall, R. L. (1998). The Plant Journal, 14(6), 733–741.) for which we changed the text from myosin VIII to just myosin.

3) l. 57-68 - The statement that "in general, the presence of F-actin at PD appears to limit symplastic movement" is an oversimplification, contradicted, e.g., by https://doi.org/10.1007/s00709-010-0244-3. A more balanced discussion of Ref. 18 is needed, such as, e.g., in https://doi.org/10.3389/fpls.2021.647123.

Response: We agree this was an oversimplification. We have modified the text to include the observations that results are variable between species, cell type, and experimental method. And we added the caveat that global depolymerization of actin does not specifically address plasmodesmata-associated actin.

4) In the Discussion, the authors should explicitely acknowledge that similar observations are narratively reported as "data not shown" in Ref. 32.

Response: Thank you for the detail. We carefully went through reference 32 (now reference 37; Dyachok et al 2008), which says “data not shown” 28 times, but none of them seemed to match with respect to our similar observations. What part of this discussion are you referring to?

5) l. 179 - it would be good to include information on light source type.

Response: Done.

6) l. 200-201 - since no statistical analyses are reported, there is no point describing their methodology!

Response: Good point. We changed the text to describe how we made the graphs.

# Reviewer #2:

This paper reports a single key finding: BRK1-YFP localizes to plasma membrane puncta. The location of these puncta correlate with a depletion of propidium iodide and deposition of callose, which mark plasmodesmata. Therefore, it is likely that BRK1-YFP localizes to plasmodesmata. BRK1 is a member of a multi-subunit complex, the SCAR/WAVE complex. While other members of the SCAR/WAVE complex have known functions outside the complex, the only known function of BRK1 is within SCAR/WAVE. The function of SCAR/WAVE is to promote actin nucleation, via ARP2/3 dependent and independent mechanisms. BRK1 itself has not been directly shown to localize to plasmodesmata, nor (to my knowledge) have other SCAR/WAVE complex members, therefore making the data presented here new. Despite the novelty of this data, it is not surprising, given that members of the ARP2/3 complex have already been shown to localize to plasmodesmata.

I am a bit ambivalent about the data in this paper. The imaging of the single BRK1-YFP transgenic is robust, however only one event has been examined, and there are no orthogonal data to support the observation. Colocalization is shown with aniline blue (but not with PLDP1). I am inclined to believe that BRK1-YFP is enriched at plasmodesmal sites, largely in part because so many other actin-associated proteins are there. If this was the first report of any SCAR/WAVE or ARP2/3 complex member localizing to PD, I would insist on either multiple transgenic events, and/or an independent line of evidence such as a native antibody, or interaction assays with other PD proteins. No functional relevance to this correlation in localization is investigated. The authors are generally cautious in their language, but none-the-less suggest that (lines 149-150) “BRK1-YFP accumulation at PD is most evident in mature cells, suggesting a specialized function in modulating secondary PD formation or regulating PD permeability (1,33).” There is no evidence provided here, or elsewhere, that BRK1 or SCAR/WAVE functions to regulate PD formation or permeability. Using the brk1 mutant vs brk1; BRK1-YFP complemented line to assay permeability using a transient transformation assay would suggest a functional role. I believe this is a relatively simple assay. Similarly, quantifying secondary PD in wildtype vs brk1 would address the question of formation, but I think this is a more difficult question, as it would likely require TEM. While normally I am reluctant to ask for additional experiments, I feel like the single result here warrants either some sort of orthogonal support, and/or some of the discussion must be toned down even more.

Response: These are great points, and we totally agree that some functional experiments like the ones suggested would strengthen the paper, but the first author is no longer at Usask, so this project is currently on hold. We have clarified and toned down this statement to indicate that BRK1-YFP localization to plasmodesmata was detectable only when pit fields are visible (via reduced PI staining along anticlinal walls), which, in our hands, tends to be in fully differentiated cells, such as the large hypocotyl cells and fully lobed pavement cells shown in figure 2.

Regarding data presentation – single channel images should be shown in grey scale to improve visibility in all figures. Green/red combinations are not color blind friendly, and should be adjusted accordingly. It would help readability considerably to (1) use consistent colors for the same construct between figures (e.g, BRK1-YFP is cyan in Figure 1, green in Figure 2, yellow in Figure 4, and green again in Figure 5.). I would also recommend that every panel be labelled with the fluorochrome examined, as in Figure 2. In my initial scan of the paper, I believed Figure 3 (which is PLDP1-GFP) showed BRK1-YFP. The white lines in Figure 1 that mark periclinal/anticlinal edges are small and hard to notice.

Response: Thank you for the feedback. We have changed the color schemes for all figures for contrast and consistent color-blind friendly styles. There are still a couple of variations in color formatting, but as suggested, we added text to each image panel to indicate the fluorophore, and we re-drew annotations in clearly contrasted colors.
